# From Simulation to Inspiration: Using Virtual Reality to Explore Rural Health Careers

**DOI:** 10.1111/ajr.70240

**Published:** 2026-08-17

**Authors:** Tegan Podubinski, Kristen Glenister, Hasan Ferdous, Muhammad Ashad Kabir, Eme John, Uchechukwu Levi Osuagwu

**Affiliations:** ^1^ Department of Rural Health The University of Melbourne Wangaratta Victoria Australia; ^2^ Centre for Digital Transformation of Health The University of Melbourne Parkville Victoria Australia; ^3^ School of Computing, Mathematics and Engineering Charles Sturt University Bathurst New South Wales Australia; ^4^ Bathurst Rural Clinical School Western Sydney University Bathurst New South Wales Australia

## Abstract

**Objective:**

To examine whether a VR rural health experience, co‐created using an established process and research protocol, could achieve similar impacts on rural practice intent and reflective engagement in a different geographic and organisational context.

**Setting:**

Metropolitan Sydney and Central Tablelands of New South Wales, Australia.

**Participants:**

Forty‐one health science students and professionals recruited at careers‐related events or while on placement. Most participants (81.1%) were from metropolitan areas, and 39% had no prior rural work exposure.

**Design:**

A parallel convergent mixed‐methods study. Participants completed pre‐ and post‐viewing surveys assessing intended practice location and presence in the VR environment. A subset (*n* = 38) participated in semi‐structured interviews exploring their experiences. Quantitative data were analysed descriptively and using Wilcoxon Signed Rank tests; interviews underwent reflexive thematic analysis.

**Results:**

Post‐VR, there was a significant shift in intended practice location (*p* = 0.035, *r* = 0.233), with increased interest in inner regional and small rural communities. Participants reported high levels of presence and engagement. Three themes and four subthemes were developed, summarised as: (1) VR as immersive and convenient, though limited in interactivity; (2) a rural education and awareness tool that contextualised the rural environment and challenged assumptions; and (3) a career decision‐making aid that reduced uncertainty, supported exploration and helped assess person–environment fit.

**Conclusion:**

A locally tailored VR rural health experience reproduced, and built on the key outcomes of an earlier study in a different context, supporting its adaptability and potential as a scalable tool for rural workforce development through critical reflection and informed career exploration.

## Abstract (highlights)

**What is already known on this subject:**

- Rural health workforce shortages persist globally, with in‐person rural placements shown to increase students' intent to practise rurally. However, these placements are resource‐intensive, not universally accessible and often shaped by metro‐centric training environments.
- Social Cognitive Career Theory highlights the need for repeated, meaningful exposures to rural contexts, yet most health education occurs in metropolitan universities, limiting opportunities for rural immersion.
- While VR can enhance engagement in higher education, evidence for its effectiveness as an educational and career intervention to support rural workforce development has been limited and context specific.

**What this study adds:**

- A co‐created VR rural health experience significantly increased participants' intent to practise in regional and rural communities.
- VR provided realistic, scalable exposure to rural work and lifestyle, supporting informed, reflective career decision‐making.
- VR challenged stereotypes and deficit views of rural practice, prompting critical reflection and reframing of assumptions.

## 1 Introduction

Rural health workforce shortages remain a critical challenge for healthcare systems globally, contributing to persistent disparities in healthcare access between rural and metropolitan areas [1]. Real‐life rural health experiences, such as student placements and training programs, have long been implemented as a strategy to address these shortages, with the aim of exposing future rural health practitioners to the value of rural practice and life [2]. These experiences provide transformative learning opportunities, allowing health students to not only develop their clinical skills and knowledge, but also to engage with rural communities, challenge their assumptions and biases and develop a deeper understanding of the unique aspects of rural healthcare [3, 4]. They also allow students to explore whether they are a good match for a rural career [5]. Importantly, exposure to rural practice has been shown to increase students' rural intent, regardless of their initial rural or metropolitan background [6]. For the purposes of this paper, the term rural encompasses all areas outside Australia's major cities [7].

Despite their demonstrated impact, not every health science student will have the opportunity to undertake an in‐person rural experience, potentially missing out on valuable exposure that could shape their interest in pursuing a rural health career. For example, one study of 268 Australian domestic students who had graduated from podiatry, occupational therapy and physiotherapy programs at the University of South Australia in 2019 found that only 40% had completed a rural placement [8]. Multiple factors are likely to contribute to this limited access, including the associated resource demands [2, 9]. For students, undertaking a rural placement often entails paying for temporary accommodation and travel, maintaining a residence in their usual location and managing lost income from time away from employment. On the provider side, successful rural placements depend on universities being supportive of such opportunities, being able to build and sustain partnerships with rural communities and facilitating access to subsidised accommodation for students. They also require the capacity of healthcare services to support student placements, especially through the availability of qualified supervisors. Collectively these barriers constrain the scalability and accessibility of in‐person rural placements.

Social Cognitive Career Theory (SCCT) provides a useful framework for understanding how careers develop, suggesting that an individual's beliefs about their own capabilities and the expected outcomes of career paths influence their choices, and that these beliefs are shaped and reinforced through meaningful learning experiences over time [10]. In the context of rural health, SCCT highlights repeated, reinforcing exposures to rural practice in cultivating interest and intent. While a single rural placement may increase initial interest, evidence suggests a cumulative effect, whereby multiple or longer‐duration rural experiences have a more significant impact on students' intentions to practise rurally [11]. Furthermore, SCCT emphasises that learning experiences extend beyond formal placements, including broader curricular activities, institutional messaging and environmental factors. Most health science courses are delivered in metropolitan universities, meaning students are often immersed in urban‐centric training environments that may not prioritise or normalise rural practice as a career goal [12, 13]. As such, the learning opportunities and professional pathways promoted within these environments shape the exposures available to students, and therefore, their beliefs about what is possible, valuable or desirable. To build a sustainable rural workforce, consideration needs to be given to not just the quantity of rural placements offered, but also how learning environments and institutional culture and curricula collectively influence career development.

Given these limitations in placement access and the influence of metro‐centric learning environments, there is a need to explore alternative, scalable approaches that can provide repeated, meaningful exposure to rural health. One such approach is to use Virtual Reality (VR) to create immersive rural health experiences that expose health students to the unique realities of life in rural communities and rural healthcare in a scalable and cost‐effective way. In the broader higher‐education context, VR experiences have been shown to increase engagement and affect positive and negative emotions [14, 15], with positive affect being especially relevant to career decision‐making [16]. A key factor influencing these outcomes is *presence* (the subjective sense of ‘being there’ in the virtual environment), which is widely recognised as a critical determinant of VR experience quality and has been positively associated with learning effectiveness [17]. Through various mechanisms, this feeling of presence can heighten emotional and cognitive engagement [18, 19], making VR a potentially powerful tool for learning. However, despite this promise, most applications of VR in higher education have focused on technical or procedural skills, with relatively limited attention to VR's role in shaping career trajectories or fostering the kinds of transformative learning experiences that underpin rural workforce development.

In our previous research, we co‐created a VR experience in partnership with a hospital in rural Northeast Victoria, showcasing the professional and lifestyle aspects of rural health, both of which are key factors in attracting a rural health workforce [20]. Our research showed that this experience helped address common misconceptions about rural practice, increased confidence in undertaking rural placements, reinforced previous positive rural experiences and ultimately led to a shift in participants' intent to practise in more rural settings [21]. While the original aim of this study was to highlight the appeal of rural health practice and increase intent to practise rurally, the results suggest that the VR experience was able to encourage participants to reflect on, and in some cases reassess, their existing beliefs and assumptions about rural practice. This kind of critical reflection is consistent with the early stages of transformative learning, a process that involves critically examining one's worldview over time to develop a more informed, inclusive and adaptive perspective [22]. Transformative learning has been increasingly recognised as valuable within rural placement experiences [13], though realising its fuller potential is understood to require sustained and repeated engagement. Consistent with SCCT this highlights the need for multiple opportunities to support sustained transformation, ensuring that learners do not revert to their previous frames of reference when returning to their usual learning environments [13, 23].

While the use of co‐developed VR to deliver immersive rural health experiences is showing promise, its effectiveness across different rural contexts remains unclear. Rural healthcare is not monolithic, and experiences vary significantly by place, population and local workforce needs [24], meaning that strategies successful in one region may not translate seamlessly to another. To explore the broader applicability and transferability of our approach, we replicated our VR intervention in a new rural context, co‐developing a second immersive VR experience in collaboration with a health service in the Central Tablelands of New South Wales (NSW), Australia. The current study aimed to build on and extend the findings of our original research. Specifically, it sought to test whether similar impacts on rural intent and reflective engagement could be achieved in a different geographic and organisational setting, using the same co‐created process and research protocol. Conducting the study through a different research team further allowed us to evaluate the robustness and reproducibility of the intervention across independent implementation. Overall, the current study contributes to a growing evidence base for VR as a scalable, context‐sensitive tool to strengthen rural health workforce development and recruitment.

## 2 Method

### 2.1 Ethics Approval and Consent

This research received ethics approval from The University of Melbourne (Project ID: 26916, June 2023). Other participating universities (Western Sydney University and Charles Sturt University) obtained ethical approval or registration from their own institutions, as appropriate. Permission to record and use footage of health workers undertaking work‐related activities, as well as to capture scenes from local events and public locations, was granted by hospital management and the regional council.

### 2.2 Development of the Virtual Reality Experience

The VR experience was developed through a collaborative co‐creation process involving the research team, a health service in the Central Tablelands of NSW, health service clinicians and management, regional council representatives and a professional videographer. The objective was to create an immersive and authentic representation of living and working in a regional Australian community, with a particular focus on factors that may influence healthcare professionals when considering rural or regional practice [20]. Content development was informed by consultations with local health professionals from a range of clinical disciplines and service areas to ensure that the VR experience accurately reflected the realities of professional practice in the Central Tablelands of NSW. Through this co‐creation process, clinicians identified the people, places, experiences and aspects of practice they considered important to showcase, providing prospective rural health professionals with an authentic insight into regional healthcare careers. Emphasis was placed on presenting a strengths‐based and experiential portrayal of both the workplace and community, while maintaining authenticity and realism. Unlike our previous study [21], which utilised researcher‐generated recordings, the current project employed a professional videographer to capture and produce the VR content. Filming was undertaken using commercially available 360‐degree video technology, and the final product was viewed using Meta Quest 2 VR headsets.

The VR experience consisted of a series of themed immersive video segments and took 30–45 min to complete. The workplace component aimed to showcase the diversity of healthcare career opportunities available within the regional health service. Filming captured a broad range of healthcare settings and professions, including Community Health services (Child and Family Health, Speech Pathology, Aboriginal Health, Ambulatory Care, Hospital in the Home and Community Nursing), Allied Health services (Occupational Therapy, Community Rehabilitation, Physiotherapy, Dietetics and Drug and Alcohol Services), Emergency Department, Intensive Care Unit, Medical and Surgical Wards, Paediatrics, Operating Theatre, Maternity Services, Oncology, Integrated Care, Nurse Practitioner services, Administration, Catering and other support services. The VR experience also featured medical students undertaking clinical placements to demonstrate opportunities for learning and professional development within the regional setting. To further highlight career pathways and professional satisfaction in regional practice, interviews were conducted with prominent local healthcare providers, including a community pharmacist and a general practitioner. These interviews explored aspects of their work they found professionally rewarding, factors contributing to career satisfaction and motivations for continuing to practise in a regional community.

The community and lifestyle component of the VR experience was designed to address non‐work‐related factors that are often important in relocation decisions. With permission from the regional council and participating organisations, filming included local historical landmarks, parks and recreational facilities, sporting and outdoor activities, educational institutions (including childcare centres, primary schools, secondary schools and university facilities), restaurants, cafés, entertainment venues and examples of local industries and employment opportunities relevant to spouses, partners and other household members.

After filming, the videographer curated the footage into a series of thematic segments covering healthcare practice, professional development opportunities, family life, education, recreation and community engagement. A narrated voice‐over was incorporated throughout the VR experience to provide context, guide viewers through the content and highlight key features of living and working in the Central Tablelands of NSW.

### 2.3 Participants and Recruitment

Health science students and professionals residing in Australia were eligible to participate and were recruited at careers related events in metropolitan Sydney or while on placement in the Central Tablelands of NSW. The VR experience was promoted in conjunction with the event, allowing interested individuals to book a specific time to participate in the research activities. Prior to participation, written informed consent was obtained from all participants.

### 2.4 Data Collection

This study utilised a parallel convergent mixed methods design to concurrently collect quantitative and qualitative data using a pre‐ and post‐survey, and semi‐structured interview (conducted by ULO and EJ). Participants were asked to complete a brief survey before (approximately 15 min) and immediately after (approximately 15 min) viewing the VR footage (approximately 20 min). The survey included demographic questions, and questions related to rural health career decision making, presence in virtual environments [25, 26] (questions used without modification or adaption), and the VR experience (Appendix A). Participants' intended practice location was assessed using the question: *‘In which geographical location within Australia would you most like to practice*?’, with response options of major or capital city, inner regional or large town, outer regional or smaller town, small rural community, remote community, and very remote community. A subset of participants took part in a brief semi‐structured interview focused on exploring their perceptions of the VR experience, which was audio recorded (approximately 5 min; Appendix B).

### 2.5 Analysis

#### 2.5.1 Quantitative Analysis

Demographic data, intended geographical location of practice, and the presence scale data from the pre‐ and post‐surveys are used in this paper. Quantitative data were analysed using a combination of descriptive and inferential statistics. For descriptive analysis, categorical variables are presented as frequency (n) and percent (%), while continuous variables are presented as mean (M) and standard deviation (SD). Intended practice location was measured as an ordinal variable, scored as 1 = major or capital city, 2 = inner regional or large town, 3 = outer regional or smaller town, 4 = small rural community, 5 = remote community and 6 = very remote community, with higher scores indicating a greater preference for rural or remote practice locations. Change in intended practice location was calculated as the within‐participant difference between post‐ and pre‐intervention scores, with positive values indicating a shift towards more rural practice locations and negative values indicating a shift towards less rural locations. Given the paired ordinal nature of these data, changes in intended practice location were analysed using the non‐parametric Wilcoxon Signed Rank test. Presence in virtual environments was analysed at the individual item level, with higher scores indicating a greater sense of presence.

#### 2.5.2 Qualitative Analysis

##### 2.5.2.1 Positionality Statement

The multidisciplinary research team included researchers with expertise in psychology, rural health, digital health, medical informatics, virtual reality, human factors and computing. Team members held differing relationships with rural Australia, including living and working in regional communities, undertaking rural health workforce research and developing technology‐enabled educational interventions. The team recognised that these experiences, particularly prior involvement in VR‐based interventions, could create an expectation that VR would positively influence perceptions of rural practice, while rural health workforce experience could equally foster scepticism that a technology‐based intervention could address deeper structural barriers to rural practice. During data collection, interviewers followed a semi‐structured interview guide and used neutral prompts to encourage participants to discuss both positive and negative aspects of the VR experience, while remaining mindful that interviews conducted immediately after the experience may have encouraged favourable or recency‐influenced responses. The competing assumptions were managed throughout analysis through reflexive discussion and cross‐disciplinary sense‐checking, as detailed below.

##### 2.5.2.2 Analysis

The interview data underwent an iterative process of reflexive thematic analysis, employing both inductive and deductive approaches. The analysis was guided by Braun and Clarke's six‐phase framework for thematic analysis [27], as well as the approach outlined by Tulip et al. [28]. Initially interviews were transcribed using Otter.ai [29] and subsequently verified for accuracy by the researchers (TP and EJ). Transcripts were reviewed multiple times, with initial codes documented (TP). The transcripts were then imported into NVivo, where coding was systematically conducted. Codes were organised into preliminary sub‐themes and later refined into overarching themes as the analysis progressed. These themes and sub‐themes were continuously reviewed and refined to ensure conceptual clarity and coherence (TP). Throughout this process, TP discussed theme development and interpretations with KG, critically examining the assumptions and biases outlined in the previous paragraph and exploring alternative explanations. All researchers reviewed the final themes and sub‐themes once stabilised, providing additional sense checking informed by their diverse disciplinary expertise and differing relationships to rural and regional Australia and to VR‐based interventions. Verbatim quotations were incorporated to illustrate the identified themes. Findings from the qualitative and quantitative analyses were synthesised in the discussion [30] and contextualised in relation to the research questions and relevant literature.

## 3 Results

### 3.1 Quantitative Results

#### 3.1.1 Demographic Details

Table 1 presents the demographic characteristics of the 41 participants in this study. Most participants were female (53.7%), currently studying (90.2%) and from the medical sector (75.6%). The majority lived in metropolitan areas (81.1%), with only one third having a rural background (34.1%). Additionally, less than half had experienced a rural placement (39.0%), rural work experience (26.8%), or rural employment as a qualified health practitioner (17.1%). Sixteen people (39%) had no exposure to student placements nor work experience nor work in a rural area.

**TABLE 1:** Characteristics of participants (*n* = 41).

Age (mean ± SD)	24.07 ± 8.83
Gender, *n* (%)	
Male	19 (46.3)
Female	22 (53.7)
Student status, *n* (%)	
Currently studying	37 (90.2)
Currently not studying	4 (9.8)
Career stage	
Early career/studying (less than 10 years' experience)	38 (92.7)
Mid‐career (10–20 years' experience)	1 (2.4)
Late career (more than 20 years' experience)	2 (4.9)
Discipline, *n* (%)	
Medicine	31 (75.6)
Nursing	5 (12.2)
Allied health, Aboriginal Health, Veterinary, Vocational education	5 (12.2)
Residential location, *n* (%)	
Metropolitan areas (MM1)	30 (81.1)
Rural, regional, remote (MM2‐7)	7 (18.9)
Rural origin, *n* (%)	
Yes	14 (34.1)
No	27 (65.9)
Previous rural student placement, *n* (%)	
Yes	16 (39.0)
No	25 (61.0)
Previous rural work experience, *n* (%)	
Yes	11 (26.8)
No	30 (73.2)
Previous rural work, *n* (%)	
Yes	7 (17.1)
No	34 (82.9)

#### 3.1.2 Rural Intent

A Wilcoxon Signed Rank Test revealed a statistically significant change in the geographical location of intended practice following viewing of the VR experience (𝑧 = −2.11, 𝑛 = 41, 𝑝 =0.035), with a small effect size (𝑟 =0.233). The geographical location of intended practice shifted after viewing the VR experience, with a decrease in preference for practicing in major cities and an increase in interest in practicing in inner regional and small rural communities (Table 2). Specifically, 17% of participants (*n* = 7) showed a shift towards a more rural intended practice location, 80% of participants showed no shift (*n* = 33) and 2% showed a shift towards more metropolitan intended practice locations (*n* = 1).

**TABLE 2:** Change in intended geographical location of practice pre‐ and post‐VR experience.

In which geographical location within Australia would you most like to practice	Pre‐viewing, *n* (%)	Post‐viewing, *n* (%)
Major city or capital city	15 (36.6)	11 (26.8)
Inner regional city or large town	13 (31.7)	15 (36.6)
Outer regional or smaller town	11 (26.8)	11 (26.8)
Small rural community	2 (4.9)	4 (9.8)

#### 3.1.3 Presence

**FIGURE 1:**
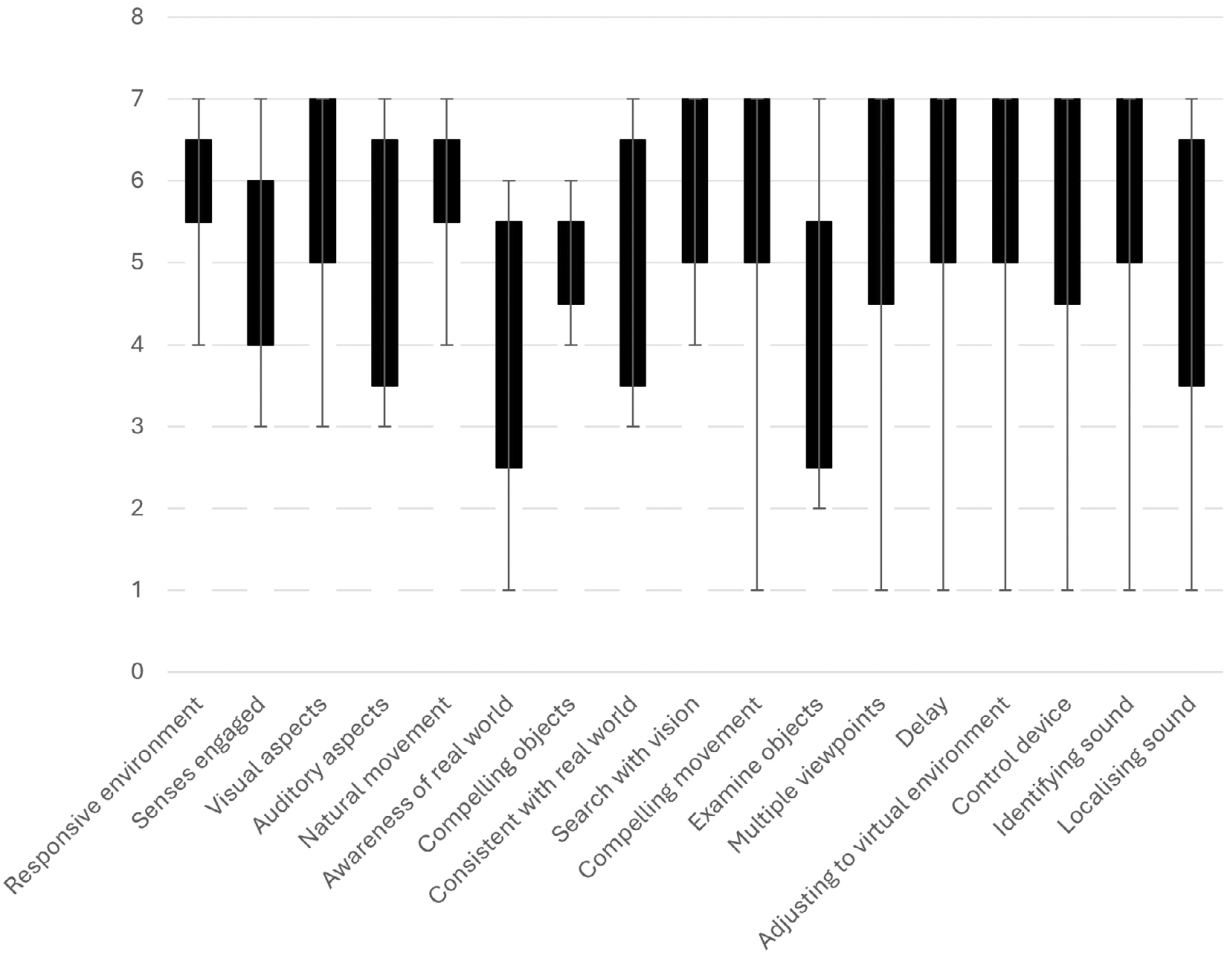
Boxplot of responses to individual components of the presence items.

Figure 1 shows a summary of the results of the presence items. There were strong positive responses to each of the individual items, except for awareness of the real world and ability to examine objects, which showed a range of positive and less positive responses.

### 3.2 Qualitative Results

A total of 38 interviews were conducted (average 5:22 min). Following reflexive thematic analysis, three key themes and four sub‐themes were developed. Themes and sub‐themes, and their associated meanings are shown in Table 3.

**TABLE 3:** Themes, sub‐themes and associated meanings.

Theme	Sub‐theme	Associated meaning
Overall perceptions of the virtual reality experience	—	VR experience as immersive, engaging and convenient. Some limited interactivity and technical issues.
A rural education and awareness tool	Contextualising rural environments	The VR experience offered a broad introduction to rural communities, allowing participants to visualise health workforce needs, professional support and aspects of the rural living.
	Challenging stereotypes and reframing perceptions	The VR experience challenged participants' preconceived stereotypes of rural areas by offering a strength‐based, immersive approach that prompted reflection and reshaped underlying assumptions and biases.
A tool in rural health career decision making	Exploring going rural	The VR experience encouraged participants to explore rural opportunities, reduced uncertainty about rural life and reinforced and deepened past rural experiences.
	Assessing for person‐environment fit	The VR experience helped participants assess whether rural practice aligned with their personal and professional needs, supporting more informed career decisions by offering a realistic preview of both rural work and lifestyle. It also raised important considerations about belonging, diversity and alternative ways to contribute to rural health.

#### 3.2.1 Overall Perceptions of the Virtual Reality Experience

Participants found the VR experience to be immersive and engaging, authentically capturing what the town was like without ‘overselling or underselling’ (Interview 6). They spoke about how convenient the experience was, allowing them to experience the town ‘without actually travelling and spending money there’ (Interview 13). While most discussed how the experience was more captivating than traditional 2D videos, a few participants described how the VR experience was not much different from traditional video on a computer:

I think the benefit of the VR headset versus just watching a video on a computer, I don't think it's that different… It was nice to, like, look around, but I think a video could show you that…. (Interview 11)

Related to this, some participants noted a desire for more interactive elements in the footage, including the ability to customise a more personalised experience and have a more interactive experience:

There wasn't the opportunity to… interactively explore and walk around… it all played out for you, like a YouTube video… The main advantage of the technology wasn't capitalised on. (Interview 17)

A small number of participants also noted usability issues, including headset discomfort, blurriness of the footage and sound quality. One participant noted some dizziness towards the end of the experience.

#### 3.2.2 A Rural Education and Awareness Tool

The interviews highlighted how the VR experience could function as an educational tool, helping participants to develop a more nuanced understanding of rural contexts. Rather than telling and describing, the immersive nature of VR allowed participants to see and engage with real‐world rural settings, offering insights into both workforce and the lifestyle experience of rural communities. Importantly, the experience fostered a strengths‐based perspective, showcasing the opportunities and unique attributes of rural life, rather than reinforcing deficit‐based narratives.

##### 3.2.2.1 Contextualising Rural Environments

Comments from the interviews highlighted how the VR experience provided a broad introduction to rural environments, encompassing factors such as professional opportunities and support, workforce demands and lifestyle considerations. This helped to develop awareness of rural workforce issues:

Yeah, I didn't realise there was a need for [occupational therapists] out in rural communities. (Interview 14)

The VR experience helped them to visualise the realities of rural health practice, enabling them to form opinions about the professional support available and the scope of interprofessional collaboration. Furthermore, it helped them to visualise various aspects of rural living, such as social connectedness and the accessibility of amenities and bigger cities:

I didn't know there was, you know, smaller towns so close to Sydney… what I thought before was they're a five‐hour drive minimum, or they're far off. (Interview 1)

##### 3.2.2.2 Challenging Stereotypes and Reframing Perceptions

A recurring theme in the interviews was the tendency for participants to enter the experience with preconceived notions of rural areas, often shaped by stereotypes of rural towns being vast, isolated places where nothing happens and opportunities are limited:

Because the assumption for me was, it's a very small town. There's nothing around, just a lot of land around. (Interview 23)

However, the VR experience played a key role in challenging and reshaping these perceptions. As one participant noted, it allowed them to ‘*challenge… the stereotypes that we hold [about rural]*’ (Interview 3). This shift was achieved not through direct instruction, but through a strength‐based, experiential approach. Further, the visual elements of the VR experience effectively demonstrated the variety of rural experiences:

Seeing this video, it kind of showed me like that, there are a lot of things you can do, and there's lots of experiences. (Interview 2)

The way participants constructed and deconstructed stereotypes was influenced by their prior experiences. Many compared rural settings to a metropolitan environment, reinforcing an urban‐dominant worldview. For others with previous rural experiences, the VR experience sometimes led to a reassessment of rural assumptions:

Yeah, it's changed my opinion of [rural town]. So, where I grew up, rurally, [rural town] was seen as a not so nice place. But watching that video, yeah, there's nothing wrong with it. (Interview 37)

#### 3.2.3 A Tool in Rural Health Career Decision‐Making

The VR experience played a role in participants' decision‐making about pursuing rural opportunities, though its impact varied based on prior experiences, career stage and existing attitudes towards rural work. Rather than acting as a single decisive factor, the VR experience functioned as a supplementary tool, helping participants explore, validate, or refine their perceptions of rural practice.

##### 3.2.3.1 Exploring Going Rural

For some participants, the VR experience acted as a starting point for exploring rural opportunities, allowing them to consider rural placements and short‐term opportunities:

This is useful for at least getting people to take the first step and go experience rural, right? So, for me… is this enough for me to be convinced that I want to live rurally? Maybe not. But is it enough that I want to try it out, you know, maybe go on the next car racing event that happens, or go volunteer for a weekend, or even longer, like a four‐week, six‐month rotation, yeah? Like, I feel like that this is enough for me to sort of do that. (Interview 23)

For others, the VR experience helped to reduce uncertainty about various aspects of rural communities, including social and professional opportunities. A key concern for many participants was whether a rural health experience would offer sufficient social interaction and connection, with the VR experience providing reassurance that rural communities are vibrant and well‐connected:

Before the video I was a little bit like, apprehensive about, like, the social opportunities, but I think it seems like there's a lot out there, and everyone puts a lot of emphasis on it. So that was the only thing that made me not want to go rural. But I think, after the video, I think it's not that bad. (Interview 9)

Many participants talked about the VR experience enhancing their real‐life rural experiences. For those who had previously visited or worked in rural areas, the experience added depth to their understanding, reinforcing positive aspects and addressing gaps in their knowledge:

And even having gone rural, it's still impactful… Already I am aware of it being really good. This added to it… It made it slightly more positive I would say. (Interview 22)

##### 3.2.3.2 Assessing for Person‐Environment Fit

Participants spoke about how the VR experience helped them to assess whether a rural environment aligned with their personal and professional needs. By providing a realistic preview, the experience allowed participants to ‘screen in’ or ‘screen out’ of rural opportunities before making any commitments. For some, the VR experience strengthened their interest in rural practice by highlighting aspects that appealed to them. For others, it confirmed that a rural career might not be the right fit, helping them make a more informed decision early in their career exploration:

It helps to imagine what your life could be like there without having to go physically to the place. (Interview 38)

Some participants mentioned the importance of showing not just the professional aspects of rural health, but also the realities of rural living. As one participant noted, when relocating for work or study, you aren't just choosing a job, you are choosing a community:

I also liked that it showed the actual town itself, not just the hospital, because I think people forget when you're relocating for work or study, you're also relocating to live and kind of live in a good way. (Interview 15)

While some participants felt that they could achieve a good fit with the rural lifestyle and community showcased in the VR experience, several participants spoke about having concerns regarding whether they would feel included or find a sense of belonging in a rural community. For these participants, the lack of visible cultural diversity in the rural town raised concerns about representation and inclusivity:

I guess I've always been a bit hesitant about the move and what I'd be missing out on in terms of lifestyle and whether, like, the environment would be accepting for me or not, like lack of a better way to put it, I've always imagined rural Australia to be very white centric. Like, I have a lot of friends here who are like, obviously, I have friends who are white, friends who are not white. A lot of my mates are from an Indian background or from, like, an Asian background. I feel like I wouldn't be able to make as many friends like that while I was in [rural town], not necessarily like friends from, like I said, I just feel a bit more uncomfortable in environments, because I feel like I'm a bit more of an outsider. (Interview 16)

Interestingly, one participant who had decided they didn't want to live rurally still had a strong interest in contributing to rural health. This comment highlighted the broad ways in which people can contribute to the rural health workforce and rural health needs, beyond moving to an environment that might not be the right fit for them:

Well, I didn't see myself working completely rurally, but I thought it's something I can see where I am still involved in some way… maybe I'm connected with people in the workforce, sort of communicating with them and trying to work with them. (Interview 27)

## 4 Discussion

This study builds upon previous research [21] exploring the use of VR to promote rural health careers, by implementing the approach in a different rural setting and health service, allowing for an assessment of its transferability and effectiveness in a new context. Consistent with earlier findings, participants demonstrated a significant shift in intended practice location following the VR experience, with increased interest in practicing in inner regional and small rural communities. Findings relating to presence were also consistent with previous research, indicating that participants experienced a strong sense of being situated within the rural town and healthcare environment despite being physically elsewhere.

The qualitative findings provide context for these quantitative outcomes. Participants described the experience as reducing uncertainty, reinforcing previous positive rural experiences and prompting some to reconsider their assumptions about rural practice; however, they also recognised that it was unlikely to determine career choice on its own. This aligns with the modest but significant shift in intended practice location, with 17% of participants shifting towards more rural preferences. Rather than functioning as a standalone recruitment intervention, the VR experience appeared to operate as an additional exposure opportunity that supported more informed consideration of rural careers. This interpretation is consistent with SCCT, which emphasises that career decisions develop through repeated learning experiences that shape perceptions of available options, expected outcomes and confidence in pursuing particular pathways [10].

Similarly, although participants remained aware of their physical surroundings while using the VR headset, interviews indicated that this did not diminish their sense of immersion or authenticity. Participants described feeling transported into the rural environment and valued the opportunity to experience aspects of rural life and practice that may otherwise be difficult to access. The interviews also provide insight into the comparatively lower presence scores relating to interactivity, with some participants expressing a desire for greater opportunities to explore the environment and interact with people and objects rather than experiencing the content passively. Thus, while the current VR experience successfully created a strong sense of place, future iterations incorporating greater user control and interaction may further enhance engagement and presence.

By achieving broadly consistent outcomes across two distinct rural contexts, and through using a locally co‐produced process, this study provides early evidence that the approach can be adapted across settings while maintaining similar outcomes. This supports the potential of co‐created VR experiences as a flexible approach for rural health workforce development, while recognising that further research is needed to evaluate longer‐term workforce impacts and implementation at scale. This aligns with calls in the rural health literature for approaches that maintain sensitivity to local contexts while also generating transferable knowledge across communities [31].

A notable point of difference in the current study was the use of professionally filmed and edited VR content, in contrast to the researcher‐produced material used in the previous study [21]. Despite this enhancement in production quality, participants across both studies reported similar levels of engagement, learning outcomes and minor technical issues, suggesting that professional production did not substantially improve the overall user experience. Interestingly, participants in the earlier study did not identify the amateur videography as a limitation; rather, some perceived it as contributing to a sense of authenticity. These findings suggest that the effectiveness of VR experiences may be influenced less by production quality alone and more by the authenticity, relevance and local ownership of the content. While the cost of video equipment and VR headsets represents a modest investment, the primary barriers for rural communities and health services to develop their own content are more likely to be time, capacity and the need for upskilling, rather than financial expense. With appropriate support and training, locally co‐produced VR experiences may therefore represent an achievable approach for rural communities seeking to develop contextually relevant workforce initiatives.

### 4.1 Reflection, Reframing and the Potential for Transformative Learning

Mezirow's theory of transformative learning emphasises the importance of critical reflection and discourse for reshaping one's worldview and fostering more inclusive and adaptive understandings [22]. Central to this process are *disorienting dilemmas*, which can be thought of as experiences that present new or conflicting information and prompt individuals to question their existing values, beliefs, or assumptions [32]. These dilemmas often lead to strong emotions, critical self‐reflection and ultimately an openness to alternative meanings or viewpoints, creating space for new knowledge to be explored and constructed [33]. However, transformative learning is generally understood as an extended process involving sustained reflection, dialogue and integration of revised perspectives into future action [22, 23, 34].

The qualitative findings suggest that participants engaged with some early processes associated with transformative learning, rather than demonstrating transformative learning in its fullest sense. Many participants described entering the VR experience with pre‐existing, often stereotyped, assumptions about rural areas as isolated, limited, or lacking opportunity. For some participants, the VR experience appeared to function as a disorienting dilemma by presenting a strengths‐based and experiential counter‐narrative that challenged these assumptions. Participants described reassessing perceptions of specific rural towns, recognising the proximity and vibrancy of rural communities, and developing a more nuanced understanding of rural health practice. Importantly, these reflections extended beyond simply receiving new information; participants actively compared the experience with their existing assumptions and reconsidered the meaning they attached to rural environments.

However, these reassessments should be interpreted as initial reflective responses rather than evidence of completed transformative learning. There was limited evidence that participants had integrated revised perspectives into broader changes in professional identity, future action, or ongoing discourse with others, which are elements considered central to consolidating transformative learning [22, 32, 33, 34]. Therefore, the findings are best understood as demonstrating that VR can prompt critical reflection and encourage reassessment of assumptions about rural practice and lifestyle, representing potential early stages of a transformative learning process. Such reflective disruption may nevertheless be valuable within healthcare education, where metropolitan settings are often implicitly positioned as the default or preferred environment for training and professional development [35]. Future research incorporating repeated exposure, longitudinal follow‐up, or structured reflective activities would help determine whether these initial reflections develop into more enduring changes in perspective.

### 4.2 Aiding Career‐Decision Making: Linking Findings to Social Cognitive Career Theory

SCCT proposes that career decision‐making is shaped by self‐efficacy beliefs, outcome expectations and the range of options individuals perceive as available and achievable [10]. Within this framework, meaningful career interventions aim to expand perceived career possibilities, clarify expectations about future outcomes and support confidence in pursuing particular pathways while addressing barriers that constrain choice [36].

The qualitative findings align with these SCCT mechanisms. The themes of contextualising rural environments and challenging stereotypes relate primarily to perceived career options and outcome expectations. Participants described recognising that rural communities were more accessible, connected and professionally diverse than they had previously assumed, expanding their understanding of what rural practice could involve. The subtheme of exploring going rural reflects how the VR experience reduced uncertainty and made rural opportunities feel more approachable. While self‐efficacy was not directly measured in this study, participants' descriptions suggest that the experience may have supported confidence in taking initial steps towards rural engagement by making placements, rotations, or short‐term experiences feel more attainable.

The subtheme of assessing person–environment fit represents an important extension of this interpretation. Rather than simply encouraging participants to choose rural practice, the VR experience allowed them to consider whether rural work and lifestyle aligned with their personal values, professional aspirations and desired living environment. For some participants, this strengthened interest in rural practice, while for others it clarified that relocation may not align with their preferences. Importantly, participants who did not identify rural relocation as the right fit still recognised alternative ways to contribute to rural health, illustrating how career exploration can broaden perceived pathways rather than simply directing individuals towards a single outcome. This, in turn, reflects the broader principle that all health professionals, regardless of practice location, should be equipped with the knowledge needed to work effectively with rural populations [37].

Together, these findings suggest that VR operates through multiple SCCT‐relevant mechanisms simultaneously: expanding awareness of possible career pathways, shaping expectations about rural life and work and supporting more informed assessment of person–environment fit. Rather than functioning as a persuasive recruitment tool, VR appears to support the reflective evaluation processes required for meaningful career decision‐making. This is consistent with, and extends, the broader literature on VR and career development [38].

In rural health recruitment and retention, an important consideration is not only the nature of healthcare work but also the lifestyle associated with the practice location [20]. However, because much health professional training occurs within metropolitan environments, many students and professionals have limited opportunities to meaningfully engage with rural contexts [12, 31]. The VR experience addressed this gap by providing an accessible form of rural exposure for individuals who may not otherwise be able to participate in rural experiences due to financial, geographic, or personal barriers. By enabling participants to explore both professional and lifestyle dimensions of rural practice, VR may provide an equitable complement to traditional rural immersion approaches.

### 4.3 Implications

This study demonstrates the potential of VR to complement resource‐intensive rural health in‐person initiatives. Its strengths lie in being cost‐effective, scalable, repeatable and inclusive, with adaptability across diverse rural contexts and health disciplines. Rather than replacing in‐person rural experiences, VR may provide an early exposure opportunity that prepares learners for placements, broadens awareness of rural career pathways and supports more informed decision‐making.

The findings also suggest that VR experiences may have greater educational value when combined with opportunities for reflection and discussion. Incorporating guided reflection, facilitated conversations, or follow‐up activities could strengthen the critical reflective processes identified in this study and support participants in integrating new perspectives into their professional development. Given these benefits, VR may represent a practical, evidence‐informed component of broader rural health workforce strategies.

### 4.4 Limitations and Future Research

While the findings of this study are encouraging, several limitations should be acknowledged. First, participants self‐selected into the study, and recruitment occurred at careers‐related events and during placements, contexts that likely attracted individuals who already had some interest in, or openness to, rural health careers. This raises the possibility of selection bias, whereby students or professionals who were disengaged from or resistant to rural practice were less likely to take part. As a result, the sample may not be representative of the broader health workforce population, and the observed shifts in rural intent may not generalise to less engaged or undecided individuals who could respond quite differently to the intervention. Future research should consider recruitment strategies that reach a wider cross‐section of students and professionals, including those with limited prior interest in rural practice, to better understand how VR interventions perform across a more heterogeneous population.

Second, the study assessed participants' immediate responses following a single exposure to the VR experience. Although shifts in intention and perception were observed, it remains unclear whether these changes are enduring without continued exposure. Longitudinal research is needed to evaluate the sustained impact of VR interventions on rural health perceptions and career decision‐making over time. Future work should also examine changes in self‐efficacy and outcome expectations, as conceptualised within SCCT, to better understand how VR influences the mechanisms that underpin career choice in rural health.

Third, the qualitative interviews were brief (averaging just over 5 min), which constrains the depth of reflection and analysis that could be captured. While this format allowed for efficient data collection across a larger number of participants and yielded meaningful themes, it may have limited opportunities for participants to fully articulate more nuanced or evolving reactions, or to explore contradictions in their own thinking. Longer or repeated interviews, potentially conducted after a period of reflection rather than immediately post‐viewing, may elicit deeper insight into how the VR experience influences participants' beliefs and career considerations over time.

Finally, although the VR experience was broadly well received, some participants reported minor usability issues and expressed a preference for greater interactivity. While these concerns did not appear to reduce the overall effectiveness of the experience, they point to opportunities for future refinement in VR design, particularly in enhancing user control, comfort and engagement. A small number of participants also perceived the VR experience as no different to a standard video, suggesting that individual preferences may influence how the technology is received, and that VR may not offer uniform benefits across all users.

## 5 Conclusion

This study provides further evidence that a co‐produced VR intervention can increase interest in rural health careers, challenge assumptions about rural health and lifestyles, and support more informed career decision‐making. By offering an accessible, immersive, and contextually adaptable form of rural exposure, VR may complement traditional rural immersion programs and extend meaningful exposure to those unable to participate in in‐person experiences.

The findings suggest that VR can operate as both a tool for critical reflection, prompting participants to notice and reassess assumptions about rural practice and lifestyle, and as a career exploration strategy that enables individuals to evaluate their fit with rural practice. Whether these early reflective processes develop into sustained transformative learning or longer‐term changes in career trajectory remains an important area for future research. Future studies should examine longitudinal effects on career intentions, self‐efficacy, outcome expectations and workforce outcomes to further establish the role of VR within rural health workforce strategies.

## Author Contributions

**Tegan Podubinski:** conceptualization, investigation, funding acquisition, writing – original draft, methodology, visualization, writing – review and editing, formal analysis, project administration, data curation, supervision. **Kristen Glenister:** conceptualization, investigation, funding acquisition, methodology, writing – review and editing, formal analysis, data curation, supervision, validation, visualization. **Hasan Ferdous:** conceptualization, investigation, funding acquisition, methodology, writing – review and editing, resources. **Muhammad Ashad Kabir:** funding acquisition, investigation, writing – review and editing, resources. **Eme John:** project administration, funding acquisition, writing – review and editing, resources. **Uchechukwu Levi Osuagwu:** funding acquisition, writing – review and editing, resources, project administration.

## Funding

This work was funded by a grant from the Commonwealth of Australia, represented by the Department of Health (Grant Activity 4‐DGEJZ1O/4‐CW7UT14).

## Conflicts of Interest

The authors declare no conflicts of interest.

## Data Availability

The data that support the findings of this study are not publicly available due to ethical restrictions.

## Appendix A Survey Questions

**Table:**

Question	Responses	Pre	Post
What is your age?	Free text	Yes	No
What is your gender	Male	Yes	No
	Female		
	Non‐binary		
What is your residential postcode?	Free text	Yes	No
If you are still studying, which year are you studying?	Not studying	Yes	No
	First year (undergraduate)		
	Second year (undergraduate)		
	Third year (undergraduate)		
	Fourth year (undergraduate)		
	Fifth year (undergraduate)		
	Other (undergraduate)		
	Postgraduate year one		
	Postgraduate year two		
	Postgraduate year three		
	Other (postgraduate)		
If you are a healthcare professional, how would you describe your career stage?	Early career (less than 10 years' experience)	Yes	No
	Mid‐career (10–20 years' experience)		
	Late career (more than 20 years' experience)		
Please describe the health discipline in which you are currently working or are currently studying.	Aboriginal/Torres Strait Islander Health	Yes	No
	Audiology		
	Dental Hygiene/Therapy		
	Dentistry		
	Oral Health Therapy		
	Diagnostic Radiography		
	Exercise Physiology		
	Medical Laboratory Science		
	Medicine		
	Midwifery		
	Nuclear Medicine Science		
	Nursing		
	Nursing & Midwifery		
	Nursing & Paramedicine		
	Nutrition		
	Nutrition & Dietetics		
	Occupational Therapy		
	Optometry		
	Orthotics & Prosthetics		
	Paramedicine		
	Pharmacy		
	Physiotherapy		
	Podiatry		
	Psychology		
	Public Health		
	Radiation Therapy		
	Social Work		
	Speech Pathology		
	Other (please describe)		
Did you grow up, or have you lived (10 years cumulatively or 5 years consecutively) in a regional, rural, or remote area?	Yes	Yes	No
	No		
Have you had any of the following rural training opportunities (tick all that apply)?	Student placement in a rural area	Yes	No
	Work experience in a rural area		
	Work in a rural area		
The following statements relate to your feelings about rural practice. Please rate your level of agreement with each statement on a scale of 1 (strongly disagree) to 5 (strongly agree).			
Rural practice is too hard.	5‐point Likert Scale (1 = strongly disagree to 5 = strongly agree)	Yes	Yes
I have the necessary skills to practice in a rural setting.	5‐point Likert Scale (1 = strongly disagree to 5 = strongly agree)	Yes	Yes
I get a sinking (anxious) feeling when I think of working in a rural setting.	5‐point Likert Scale (1 = strongly disagree to 5 = strongly agree)	Yes	Yes
I have a strong positive feeling when I think of working in a rural setting.	5‐point Likert Scale (1 = strongly disagree to 5 = strongly agree)	Yes	Yes
People tell me I should work in a rural setting.	5‐point Likert Scale (1 = strongly disagree to 5 = strongly agree)	Yes	Yes
I see people like me taking up rural clinical practice.	5‐point Likert Scale (1 = strongly disagree to 5 = strongly agree)	Yes	Yes
I could see myself living in a rural area.	5‐point Likert Scale (1 = strongly disagree to 5 = strongly agree)	Yes	Yes
I could learn how to be a good rural practitioner.	5‐point Likert Scale (1 = strongly disagree to 5 = strongly agree)	Yes	Yes
I am interested in living rurally.	5‐point Likert Scale (1 = strongly disagree to 5 = strongly agree)	Yes	Yes
I am interested in a rural health career.	5‐point Likert Scale (1 = strongly disagree to 5 = strongly agree)	Yes	Yes
If I work in a rural area, there will be many opportunities to improve my career.	5‐point Likert Scale (1 = strongly disagree to 5 = strongly agree)	Yes	Yes
If I work in a rural area, I will be able to practice a variety of skills.	5‐point Likert Scale (1 = strongly disagree to 5 = strongly agree)	Yes	Yes
If I worked in a rural area, I will be able to have a successful career.	5‐point Likert Scale (1 = strongly disagree to 5 = strongly agree)	Yes	Yes
If I live in a rural area, I will be able to do things I enjoy.	5‐point Likert Scale (1 = strongly disagree to 5 = strongly agree)	Yes	Yes
If I live in a rural area, I will be able to enjoy a vibrant social life.	5‐point Likert Scale (1 = strongly disagree to 5 = strongly agree)	Yes	Yes
In which geographical location within Australia would you most like to practice?	Major city or capital city	Yes	Yes
	Inner regional city or large town		
	Outer regional or smaller town		
	Small rural community		
	Remote community		
	Very remote community		
	I am not considering working within Australia		
On a scale of 1 (no previous experience) to 5 (very familiar), please indicate your current level of familiarity with Virtual Reality technology.	5‐point Likert Scale (1 = no previous experience to 5 = very familiar)	Yes	No
Thinking about the town of Bathurst (where this footage was filmed), please rate your level of agreement with the following statements on a scale of 1 (strongly disagree) to 5 (strongly agree).			
I could identify with the people in this community.	5‐point Likert Scale (1 = strongly disagree to 5 = strongly agree)	No	Yes
The landscape of this area makes me feel strong positive emotions.	5‐point Likert Scale (1 = strongly disagree to 5 = strongly agree)	No	Yes
I feel connected to this place.	5‐point Likert Scale (1 = strongly disagree to 5 = strongly agree)	No	Yes
This place reflects the type of person I am.	5‐point Likert Scale (1 = strongly disagree to 5 = strongly agree)	No	Yes
This is the best place for what I like to do.	5‐point Likert Scale (1 = strongly disagree to 5 = strongly agree)	No	Yes
I would like to spend time in this place.	5‐point Likert Scale (1 = strongly disagree to 5 = strongly agree)	No	Yes
The following questions ask about your experience with the Virtual Reality footage. Please read the responses carefully as the options change for each question. Please rate your level of agreement with the following statements on a scale of 1 (low levels) to 7 (high levels). Please consider the entire scale when making your responses, as the intermediate levels may apply.			
How responsive was the environment to actions (e.g., your movements) that you initiated (or performed)?	7‐point Likert Scale (1 = not responsive to 4 = moderately responsive to 7 = completely responsive)	No	Yes
How completely were all of your senses engaged?	7‐point Likert Scale (1 = not at all to 4 = somewhat to 7 = completely)	No	Yes
How much did the visual aspects of the environment involve you?	7‐point Likert Scale (1 = not at all to 4 = somewhat to 7 = completely)	No	Yes
How much did the auditory aspects of the environment involve you?	7‐point Likert Scale (1 = not at all to 4 = somewhat to 7 = completely)	No	Yes
How natural was the mechanism which controlled movement through the environment?	7‐point Likert Scale (1 = extremely artificial to 4 = borderline to 7 = completely natural)	No	Yes
How aware were you of events occurring in the physical world around you?	7‐point Likert Scale (1 = completely aware to 4 = somewhat aware to 7 = completely unaware)	No	Yes
How compelling was your sense of objects moving through space?	7‐point Likert Scale (1 = not at all to 4 = moderately compelling to 7 = very compelling)	No	Yes
How much did your experiences in the virtual environment seem consistent with your real‐world experiences?	7‐point Likert Scale (1 = not consistent to 4 = moderately consistent to 7 = very consistent)	No	Yes
How completely were you able to actively survey or search the environment using vision?	7‐point Likert Scale (1 = not at all to 4 = somewhat to 7 = completely)	No	Yes
How compelling was your sense of moving around inside the virtual environment?	7‐point Likert Scale (1 = not compelling to 4 = moderately compelling to 7 = very compelling)	No	Yes
How closely were you able to examine objects in the virtual reality experience?	7‐point Likert Scale (1 = not at all to 4 = pretty closely to 7 = very closely)	No	Yes
How well could you examine objects from multiple viewpoints (e.g., Moving head)?	7‐point Likert Scale (1 = not at all to 4 = somewhat to 7 = extensively)	No	Yes
How much delay did you experience between your actions and expected outcomes?	7‐point Likert Scale (1 = no delays to 4 = moderate delays to 7 = long delays)	No	Yes
How quickly did you adjust to the virtual environment experience?	7‐point Likert Scale (1 = not at all to 4 = slowly to 7 = < 1 min)	No	Yes
How much did the control devices interfere with the performance of assigned tasks or with other activities?	7‐point Likert Scale (1 = not at all to 4 = interfered somewhat to 7 = interfered greatly)	No	Yes
How well could you identify sounds?	7‐point Likert Scale (1 = not at all to 4 = somewhat to 7 = completely)	No	Yes
How well could you localise sounds?	7‐point Likert Scale (1 = not at all to 4 = somewhat to 7 = completely)	No	Yes
Please add any comments here	Free text	No	Yes

## Appendix B Semi‐Structured Interview Questions

- How did you feel about the VR simulation?
  ○ What did you like/not like?
  ○ What could be improved about the simulation?
- Do you see yourself in a rural health career?
- Do you think the VR simulation changed your thoughts about a rural health career?
  ○ Why/why not?
- Do you see yourself living rurally?
- Do you think the VR simulation changed your thoughts about rural places?
  ○ Why/why not?
